# Immunological Cross-Reactivity of an Ancestral and the Most Recent Pandemic Norovirus GII.4 Variant

**DOI:** 10.3390/v11020091

**Published:** 2019-01-22

**Authors:** Kirsi Tamminen, Maria Malm, Timo Vesikari, Vesna Blazevic

**Affiliations:** Vaccine Research Center, Faculty of Medicine and Health Technology, Tampere University, Biokatu 10, FI-33520 Tampere, Finland; kirsi.tamminen@tuni.fi (K.T.); maria.malm@tuni.fi (M.M.); timo.vesikari@tuni.fi (T.V.)

**Keywords:** norovirus, ancestor, variant, GII.4, immune responses, cross-reactivity, blocking antibodies, NERK motif

## Abstract

Norovirus (NoV) genotype GII.4 is responsible for the majority of NoV infections causing pandemics every few years. A NoV virus-like particle (VLP)-based vaccine should optimally cover the high antigenic variation within the GII.4 genotype. We compared the immune responses generated by VLPs of the ancestral GII.4 1999 strain (GII.4 1995/96 US variant) and the most recent GII.4 Sydney 2012 pandemic strains in mice. No significant differences were observed in the type-specific responses but GII.4 1999 VLPs were more potent in inducing high-avidity antibodies with better cross-reactivity. GII.4 1999 immune sera blocked binding of GII.4 2006 and GII.4 2012 VLPs to the putative receptors in a surrogate neutralization assay, whereas GII.4 2012 immune sera only had low blocking activity against GII.4 2006 VLPs. Amino acid substitution in the NERK motif (amino acids 310, 316, 484, and 493, respectively), altering the access to conserved blocking epitope F, moderately improved the cross-blocking responses against mutated GII.4 2012 VLPs (D310N). NoV GII.4 1999 VLPs, uptaken and processed by antigen-presenting cells, induced stronger interferon gamma (IFN-γ) production from mice splenocytes than GII.4 2012 VLPs. These results support the use of GII.4 1999 VLPs as a major component of a NoV vaccine.

## 1. Introduction

Norovirus (NoV) GII.4 is the predominating NoV genotype, causing up to 85% of acute gastroenteritis outbreaks of NoV and sporadic infections worldwide [1]. It is associated with more severe clinical manifestations than other NoV genotypes [2,3]. NoV genogroup II (GII) and GI together comprise of over 28 genetically divergent NoV genotypes infecting humans [4]. The predominance of GII.4 for over two decades is associated with several factors including fast replication and effective person-to-person transmission rates [5,6]. In addition, GII.4 variants recognize a wide range of mucosal polysaccharides [7] and histo-blood group antigens (HBGAs), which are thought to facilitate NoV entry and/or infection [8,9].

The NoV particle consists of 90 dimers of capsid VP1 protein organized in T = 3 icosahedral symmetry [10]. VP1 is divided into two main domains: the shell (S) and the protruding (P) domains, the latter of which is further subdivided into P1 and P2 domains [10]. The outermost P2 domain contains the conserved HBGA binding sites but the regions surrounding these sites are evolving due to constant immune pressure [11]. NoV vaccine development is largely based on NoV virus-like particles (VLPs) [12,13,14], antigenically identical to the virus particle, despite recent progress in cultivating NoV in vitro [15].

GII.4 NoV undergoes epochal evolution similar to influenza virus; periods of stasis lead into rapid antigenic drift in common structural epitopes that are under immune pressure [16,17]. NoV-specific serum antibodies that block binding of NoV VLPs to HBGAs in a surrogate neutralization assay are the best correlate of protection identified so far [18,19,20]. Antigenic drift driven by these antibodies can lead the evolving strains gaining new HBGA binding abilities and/or escaping from previously gained immunity [21,22,23]. There are at least six (A–F) evolving “blocking epitopes” described [21,23,24,25] and the emergence of a new GII.4 pandemic strain is typically associated with mutations in these epitopes [23,24,26]. Since the 1990s seven pandemics have been caused by GII.4 variants: Grimsby (1995/96 US), Farmington Hills (2002), Hunter (2004), Yerseke (2006a), Den Haag (2006b), New Orleans (NO, 2009), and Sydney (2012) [1].

Virions are dynamic structures reacting to environment with conformational changes, which enable biologically relevant functions such as receptor/ligand binding [27]. Mutations in the virion core, or neutralizing antibody binding to certain epitopes, can sterically block receptor binding site or cause conformational change in distant epitopes impairing ligand interactions [27,28,29]. Some of the highly variable blocking epitopes of NoV GII.4 are exposed on the surface of P2-domain (e.g., epitopes A and D) while others, like epitope F, are buried and broadly conserved [25]. Epitope F is considered a universal GII.4 blocking epitope, as monoclonal antibody specific to epitope F has been shown to cross-block a panel of time-ordered GII.4 VLPs [30]. Amino acids 310, 316, 484, and 493 comprise “the NERK motif”, which has been suggested to limit antibody access to epitope F [31].

In this study we investigated type-specific and cross-reactive humoral and cellular immune responses induced in mice with the first (GII.4 1999, a 1995/96 US variant) and the latest (GII.4 Sydney, 2012) pandemic GII.4 variant VLPs. In addition, we studied the effect of an amino acid mutation in the NERK motif on cross-blocking antibody responses. The results presented here add to the current knowledge and understanding of cross-reactive immune responses induced in vivo by different variant GII.4 VLPs.

## 2. Materials and Methods

### 2.1. NoV VLPs

Three different NoV GII.4 variant VLPs were used as immunogens and/or in vitro antigens in this study. GII.4 1999 (original patient sequence isolated in 1999; it has one amino acid difference (aa 333) to the VP1 sequence of the reference strain GII.4 1995/96 US, Genbank accession number AF080551) and GII.4 2012 (accession number AFV08795.1) VLPs were produced in baculovirus-insect cell system and purified as previously described by our laboratory [32,33,34]. GII.4 2006 variant VLPs (accession number BAG70446) were produced by Icon Genetics Gmbh (Halle, Germany) [35] and were utilized in analytical methods only. The epitope-engineered GII.4 2012 pFastBac1-vector with amino acid D310 substituted to N310 was ordered from GeneArt (Thermo Fisher Scientific, Waltham, MA, USA) and expressed using the Bac-to-Bac Baculovirus Expression System (Invitrogen, Carlsbad, CA, USA) as described before [32]. Mutant VLPs (referred as GII.4 2012 D310N) were produced and purified with the same methodology as the wild-type VLPs. The identity, purity, and morphology [36,37] of the VLPs were confirmed as described elsewhere [32,33,38].

### 2.2. Mouse Immunizations and Tissue Collections

Seven-week-old female BALB/c OlaHsd mice obtained from Envigo RMS BV (Horst, the Netherlands) were immunized intramuscularly (IM) twice (weeks 0 and 3) with 10 µg of GII.4 1999 (5 mice) or GII.4 2012 VLPs (4 mice). Mice receiving carrier buffer only (phosphate buffered saline, PBS) were used as negative controls (5 mice). Whole blood and lymphoid tissue were collected at the time of euthanization on study week 5. For bone marrow-derived dendritic cell (BMDC) generation, femurs and tibia were collected from naive control mice and the exterior tissues were sterilized with 70% ethanol and kept on ice until bone marrow extraction (described in Section 2.6). Serum was separated by centrifugation and splenocyte suspensions of each mice were prepared according to earlier published methods [38]. All procedures were authorized and performed in concordance with the guidelines by the Finnish National Animal Experiment Board (permission number ESAVI/10800/04.10.07/2016). 

### 2.3. IgG Titer and Avidity Assay

Antigen-specific and cross-reactive immunoglobulin G (IgG) responses in mice sera were measured by enzyme-linked immunosorbent assay (ELISA) as described in detail elsewhere [32,38]. Individual sera were added by decreasing two-fold dilutions (IgG titer determination) or 1:100 dilution (avidity assay) on 96-well half-area polystyrene plates (Corning Inc., Corning, NY, USA) coated overnight at 4 °C with NoV VLPs (1 µg/mL) and blocked for one hour at room temperature (RT) with 2% skimmed milk in PBS/0.05%Tween. After serum incubation, the bound antibodies were detected by goat anti-mouse IgG-HRP (dilution 1:6000, Sigma-Aldrich, Saint Louis, MO, USA) reacting with *o*-phenylenediamine dihydrochloride (OPD)-substrate (30 min RT). All incubations were performed at 37 °C for one hour unless otherwise stated. After stopping the substrate reaction with 2M H_2_SO_4_ the optical density (OD) was measured at 490 nm (Victor2 1420; PerkinElmer, Waltham, MA, USA). Each sample/dilution was assayed in duplicate wells and sample volumes were 50 µl/well. The background signal from wells lacking serum (blank wells) was subtracted from all of the OD readings at a plate. The cut-off value was calculated as mean OD + 3 × SD of negative control mice sera at a given dilution. A sample resulting in an OD value above the set cut-off OD and at least 0.100 OD was considered positive. End-point antibody titers were defined as the highest dilution of serum giving an OD above the set cut-off value. Geometric mean titers (GMTs) with 95% confidence intervals (CIs) for each immunization group were counted from individual mice end-point titers.

Serum IgG avidity was measured by ELISA as described above, but after serum incubation the plates were incubated twice (for 5 min for each treatment) with 8 M urea (Sigma-Aldrich) to elute low-avidity antibodies [39]. The avidity index was calculated as (OD with urea/OD without urea) × 100%.

### 2.4. Carbohydrate Binding Assays

HBGA binding of mutated GII.4 2012 D310N VLPs in comparison to wild-type GII.4 2012 VLPs was analyzed by using three different sources of HBGAs: human type A saliva [7,40], pig gastric mucin (PGM, type III, Sigma-Aldrich) [41,42], and synthetic biotinylated HBGAs (Lewis^a^, Lewis^b^, H-type-1, H-type-3, A-trimer, and B-trimer, Glycotech, Gaithersburg, MD, USA) [7,18]. Briefly, saliva (at dilution 1:3000, o/n 37 °C) or PGM (2.5 µg/mL, o/n RT) were used to coat half-area 96-well plates (Corning) and synthetic biotinylated carbohydrates (2.5 µg/mL, 1 h RT) were used on precoated NeutrAvidin plates (Pierce, Rockford, MI, USA). VLPs were added on the plates with final concentration of 0.1 µg/mL for 1.5 h (saliva and PGM plates) or 0.4 µg/mL for 2 h (synthetic HBGA plates). The bound VLPs were detected with human GII.4-positive serum following horseradish peroxidase (HRP)-conjugated anti-human IgG secondary antibody (Novex, Invitrogen) incubation (1 h). After coating, saliva plates were incubated at 37 °C, PGM plates at RT, and synthetic HBGA plates at 4 °C according to the temperatures used in the original publications [18,40,41]. The plates were developed and stopped as described in Section 2.3 for ELISA procedures. Positive reactivity is defined as a mean OD >0.2 after background subtraction. Each sample was tested in 2–4 replicate wells and results are represented as mean OD from parallel wells.

### 2.5. Blocking Assays

Blocking assay was used to measure antibodies that block binding of NoV VLPs to HBGAs present in human saliva (type A) or PGM, or to synthetic biotinylated H-type-1 carbohydrate as previously described [7,42]. NoV VLPs with final concentration of 0.1 µg/mL (saliva and PGM assays) or 0.4 µg/mL (synthetic HBGA assays) were preincubated for 1 h at 37 °C in low binding tubes with decreasing concentration of mice serum (starting at 1:100 serum dilution for homologous and 1:20 for cross-blocking assays). The pre-incubated VLP-serum mixtures were then added on saliva, PGM, or synthetic H-type-1 coated plates, and the bound VLPs were detected with human GII.4-positive serum (for GII.4 1999 and GII.4 2012 VLP detection) or rabbit NoV-hyperimmune serum (for GII.4 2006 VLP detection) following the corresponding HRP-conjugated secondary antibody incubation. The incubation times and temperatures were the same as described in Section 2.4 for VLP-binding assays. The plates were developed, stopped, and measured as described in Section 2.3. Wells incubated with VLPs lacking mouse sera were used to determine the maximum binding OD. The blocking index (%) was calculated as 100% − [(OD wells with VLP − serum mix/OD maximum binding OD) × 100%]. The blocking titer 50 (BT50) value represents the highest serum dilution blocking 50% of the maximum VLP binding. The results are expressed as the mean blocking indexes of individual mice or the mean of replicas or repeated experiments if pooled group sera were used.

### 2.6. BMDC Generation and Pulsing

The method for generating mouse BMDCs was adapted from a published procedure [43] with some modifications. After removing soft tissue, the femurs and the tibiae were cut from each end with scalpel and flushed with ice cold PBS. The extracted bone marrows were passed through a 70-µm cell strainer (Becton-Dickinson, BD, Franklin Lakes, NJ, USA) and collected in complete medium (CM, RPMI-1640 supplemented with 100 U/mL penicillin, 100 μg/mL streptomycin, 50 μm 2-mercaptoethanol, 2 mm l-glutamine, and 10% fetal bovine serum (FBS), all purchased from Sigma-Aldrich). The cell suspensions were centrifuged 300× *g* for 10 min and suspended in CM containing 20 ng/mL recombinant mouse granulocyte-macrophage colony-stimulating factor (GM-CSF, Abcam, Cambridge, UK). BM-cells were seeded at 1×10^6^ cells/mL (10 mL per plate) in non-treated 90 × 14.2-mm sterile petri dishes (VWR, Radnor, PA, US) and cultured at 37 °C, 5% CO_2_ for 8 days. Fresh CM with GM-CSF (5 mL/plate) was added on the dishes on days 4 and 7 and the cells were harvested on day 8. The generated cells were surface stained with phycoerythrin (PE)-conjugated anti-mouse CD11c and Horizon Viability Stain 780 (both from BD) and acquired using BD FACSCanto II flow cytometer as described earlier [44] which confirmed the cells to be >90% CD11c+ cells. The BMDCs were frozen according to published procedure [45] in ice-cold CM containing 10% DMSO (Sigma-Aldrich).

The BMDCs were thawed, washed twice (300× *g*, 10 min) and seeded 2×10^6^ cells/mL in CM in non-treated cell-culture 24-well plates (Corning Costar) for antigen pulsing. GII.4 1999, GII.4 2012 and GII.4 2006 VLPs were added to the cells at 100 µg/mL. Cells lacking pulsing antigen (un-pulsed BMDCs) were used as a negative control. The cells were incubated (37 °C, 5% CO_2_) for 20–22 hours, washed twice, and were used as antigen-presenting cells (APCs) in enzyme-linked immunospot (ELISPOT) assay.

### 2.7. ELISPOT-Interferon Gamma (IFN-γ)

Homologous and cross-reactive T-cell responses were analyzed by quantification of IFN-γ production from group-wise pooled mice splenocytes in response to VLP pulsed BMDCs by an ELISPOT-IFN-γ assay [36,38]. Briefly, 96-well MultiScreen HTS-IP filter plates (Millipore, Billerica, MA, USA) were coated overnight at 4 °C with anti-mouse IFN-γ (Mabtech Ab, Nacka Strand, Sweden). After washing and blocking the plates with 10% FBS in CM (2–3 h RT), GII.4 1999, GII.4 2012, GII.4 2006 VLP pulsed or un-pulsed BMDCs (5000, 20,000, and 40,000 BMDCs/well) were added on plates. Concanavalin A (ConA; Sigma-Aldrich) at 10 µg/ml was used as a positive control to stimulate IFN-γ production from splenocytes. GII.4 1999 or GII.4 2012 VLP-immunized mice splenocytes were thawed, washed, and added to the plates (0.2 × 10^6^ cells/well) on top of BMDCs and the plates were incubated for 20 h at 37 °C and 5% CO_2_. Thereafter, the cells were discarded and the plates were developed with biotinylated anti-mouse IFN-γ monoclonal antibody (0.5 μg/mL in PBS/0.5% FBS, 2 h at RT) and alkaline-phosphatase (ALP) conjugated streptavidin (1:1000, 1 h at RT) reacting with BCIP/NBT substrate (all from Mabtech). After 12 minutes the color reaction was stopped with tap water. The spots were counted by an ImmunoSpot^®^ automatic CTL analyzer (CTL-Europe GmbH, Bonn, Germany) and the results are expressed as mean spot forming cells (SFCs) per 10^6^ live splenocytes of replicate wells.

### 2.8. Statistics

The Kruskal–Wallis’ test was used to assess the statistical differences in antibody titers and avidity indices between individual immunization groups. A statistically significant difference was defined as a *p*-value of <0.05. Data were analyzed with IBM SPSS Statistics version 25.0 (SPSS Inc., Chicago, IL, USA).

## 3. Results

### 3.1. NoV GII.4 Type-Specific and Cross-Reactive IgG Antibody Titers and Avidity

**Figure 1:**
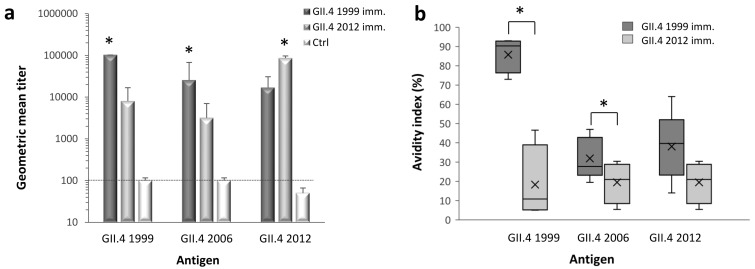
Titers and avidity of norovirus (NoV) type-specific and cross-reactive serum immunoglobulin G (IgG) antibodies. Mice were immunized with GII.4 1999 (5 mice) and GII.4 2012 (4 mice) virus-like particles (VLPs) and the immune sera was used in enzyme-linked immunosorbent assay (ELISA) to determine the magnitude of IgG antibodies against homologous and heterologous NoV VLPs (**a**). Serum of mice receiving phosphate buffered saline (PBS) (5 mice) was used as a negative control (Ctrl). Shown are the geometric mean titers (GMTs) with 95% confidence intervals (error bars) counted from individual mice end-point titers in each immunization group. The dashed line illustrates the cut-off titer for samples considered positive. The avidity of IgG antibodies was measured from individual mice sera against homologous and heterologous NoV VLPs (**b**) as described in the Material and Methods. Horizontal lines in the box plots represent the medians, cross-symbols (×) represent the means, and the boxes illustrate the interquartile range that contains 50% of values with whiskers extending to the highest and lowest values. The antigen-specific antibody titers and the avidity indexes between immunization groups were compared by the Kruskal–Wallis test and significant differences (*p* value <0.05) are identified with an asterisk (*).

Immunized mice sera were tested using ELISA to quantify type-specific and cross-reactive IgG antibodies against GII.4 1999, GII.4 2006, and GII.4 2012 VLPs (Figure 1a). GII.4 1999 and GII.4 2012 immunizations resulted in equally high magnitudes (*p* = 0.264) of type-specific IgG response, with GMTs of 102,400 and 86,100 (95% CI = 53,200–139,300), respectively. IgG responses against homologous VLPs were significantly higher (*p* < 0.05) than cross-reactive responses induced by heterologous antigen. GII.4 1999 VLP immunization induced significantly higher (*p* = 0.018) cross-reactive IgG response against GII.4 2006 VLPs (GMT 25,600, 95% CI = 9600–69,900) than GII.4 2012 VLP immunization (GMT 3200, 95% CI = 1500–7000). When GII.4 1999 and GII.4 2012 cross-reactive responses were compared against each other, GII.4 1999 immunization resulted in 2-fold higher GII.4 2012-specific titer (GMT 16,900, 95% CI = 5800–49,200) than GII.4 2012 immunization against GII.4 99 (GMT 8060, 95% CI = 3900–16,900) but the difference was not statistically significant (*p* = 0.15). Control mice did not develop specific IgG response to any of the VLPs tested.

Comparison of the avidity of type-specific and cross-reactive antibodies are shown in Figure 1b. A remarkable difference was observed in the type-specific avidities elicited by GII.4 1999 and GII.4 2012 VLPs (Figure 1b). GII.4 1999 VLPs induced type-specific IgG antibodies with high avidity (mean avidity index 85.7 ± 9%), whereas GII.4 2012 type-specific antibody avidity was poor (19.4 ± 10.7%). As expected, the avidity of GII.4 1999 immune serum against heterologous VLPs was considerably lower than type-specific avidity, but still at intermediate level (mean avidity indexes ranging from 30% to 40%). GII.4 1999 VLP immunization elicited cross-reactive antibodies against GII.4 2006 VLPs with significantly (*p* = 0.027) higher avidity than GII.4 2012 VLP immunization.

### 3.2. Blocking Antibody Responses

**Figure 2:**
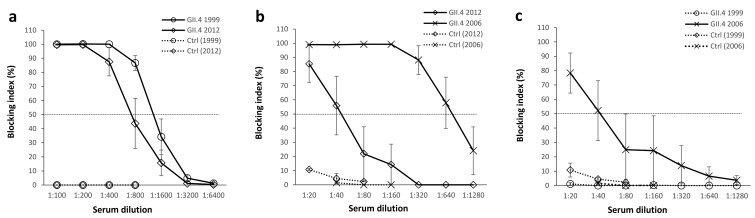
Norovirus (NoV) type-specific and cross-reactive blocking antibodies. GII.4 1999 (5 mice) or GII.4 2012 (4 mice) virus-like particle (VLP) immunized mice sera were individually diluted 2-fold starting from 1:100 dilution and assayed for the blocking of homologous NoV VLPs binding to human type A saliva (**a**). The cross-blocking activity of GII.4 1999 (**b**) and GII.4 2012 (**c**) VLP immunized mice sera as well as control mice (Ctrl, 5 mice) was assayed against heterologous VLPs as 2-fold dilution series starting from 1:20 dilution. The blocking index (percent) was calculated as follows: 100% × ((OD wells with serum/OD wells without serum, maximum binding) × 100%). The symbols represent the immunization group mean blocking indexes and the error bars represent the standard error between individual mice. The horizontal dashed line represents the blocking titer 50% (BT50).

The ability of mouse immune sera to block homologous and heterologous VLPs binding to HBGAs present in human saliva (type A) was tested in a blocking assay [7,40]. Both GII.4 1999 VLP and GII.4 2012 VLP immunizations induced strong homologous blocking activity as mean serum titers blocking at least 50% of VLP binding (BT50) were 1:800 for GII.4 1999 and 1:400 for GII.4 2012 (*p* = 0.079, Figure 2a). However, when GII.4 1999 and GII.2 2012 immune sera were used to block heterologous VLPs binding, significant differences were observed in cross-blocking responses (Figure 2b,c). GII.4 1999 VLP immunization (Figure 2b) elicited strong blocking activity against GII.4 2006 VLPs (BT50 = 1:640) while blocking against GII.4 2012 VLP binding was considerably lower (BT50 = 1:40). In contrast, GII.4 2012 immune sera (Figure 2c) blocked only GII.4 2006 VLP binding (BT50 = 1:40) at a significantly lower magnitude than GII.4 1999 immune sera (*p* = 0.036) and completely failed to block GII.4 1999 binding. Control mice sera blocked all VLP binding <15% at the lowest serum dilution (1:20).

### 3.3. Morphology, Antigenicity, and HBGA-Binding Profile of Genetically Engineered GII.4 2012 D310N VLPs

**Figure 3:**
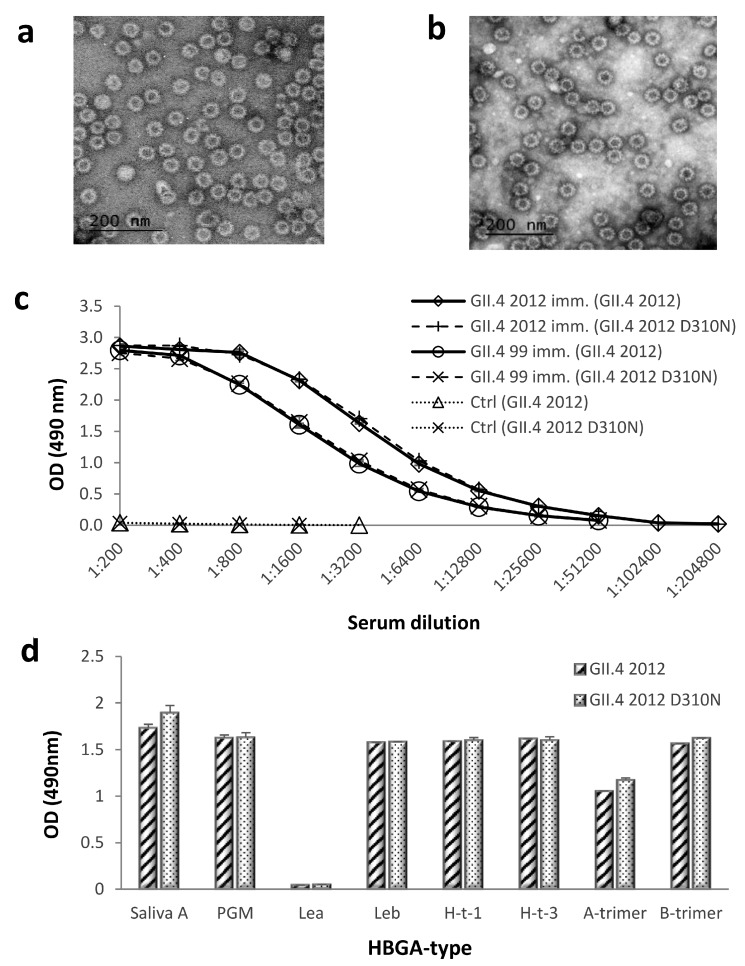
Morphology, antigenicity, and histo-blood group antigen (HBGA) binding of wild-type and mutated GII.4 2012 virus-like particles (VLPs). Morphology of GII.4 2012 D310N (**a**) and GII.4 2012 (**b**) VLPs were examined by FEI Tecnai F12 electron microscope (Philips 487 Electron Optics, Holland) after negative staining with 3% uranyl acetate pH 4.6. Mutated GII.4 2012 D310N VLPs and wild-type GII.4 2012 VLPs were used as antigens with enzyme-linked immunosorbent assay (ELISA), reacting with GII.4 2012, GII.4 1999, and control mice (ctrl) immune sera (**c**). VLPs were assayed for binding to saliva type A, pig gastric mucin (PGM), and synthetic histo-blood group antigens as described in the Materials and Methods (**d**). The symbols (**c**) and bars (**d**) illustrate the mean optical density (OD) at 490 nm of replicate wells (2–4) with standard errors. Le^a^, Lewis^a^; Le^b^, Lewis^b^; H-t-1, H-type-1; H-t-3, H-type 3.

The NERK motif (amino acids 310, 316, 484, and 493, respectively) is suggested to control antibody access to epitope F, a putative universal blocking epitope in the GII.4 lineage [31]. We genetically engineered GII.4 2012 VLP to revert amino acid 310 from D to N, as present in GII.4 1999 VLP. The morphology of GII.4 2012 D310N VLPs was studied by electron microscopy, which confirmed that the mutation did not affect mutated VLP integrity or morphology (Figure 3a) in comparison to wild type GII.4 2012 VLPs (Figure 3b). The antigenicity of GII.4 2012 D310N VLPs was further investigated using ELISA (Figure 3c). GII.4 1999 and GII.4 2012 VLP-immunized mice sera recognized wild-type (GII.4 2012) and mutated VLPs (GII.4 2012 D310N) with similar intensity (Figure 3c). HBGA binding assay against human saliva A, PGM, and synthetic carbohydrates confirmed that mutation D310N did not affect the ligand-binding abilities of the mutated VLPs (Figure 3d).

### 3.4. Blocking Antibody Responses against Genetically Engineered GII.4 2012 D310N VLPs

**Figure 4:**
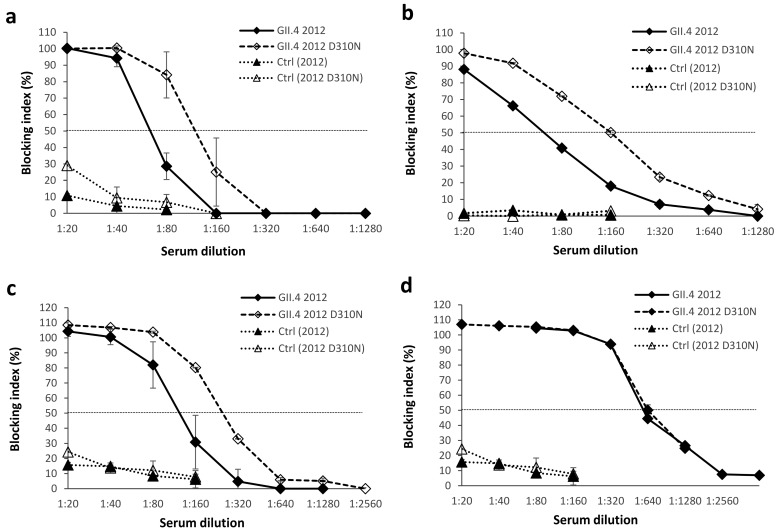
The effect of amino acid (aa) 310 mutation within the NERK motif on cross-blocking activity. Residue 310 of GII.4 2012 virus-like particles (VLPs) was substituted from D (aspartic acid) to N (asparagine) to generate mutated GII.4 2012 D310N VLPs. Pooled GII.4 1999 immune serum was assayed against wild-type and mutated GII.4 2012 VLPs in blocking assays utilizing human saliva type A (**a**), pig gastric mucin (**b**), or synthetic H-type-1 (**c**) as the source of histo-blood group antigens (HBGAs). Pooled GII.4 2012 immune serum was used to block the binding of GII.4 2012 and GII.4 2012 D310N VLPs to synthetic H-type-1 HBGAs (**d**). Control (Ctrl) mice sera illustrate the non-specific blocking activity. The blocking index (percent) was calculated as follows: 100% × ((OD wells with serum/OD wells without serum, maximum binding) × 100%). The symbols represent the mean blocking indexes with standard errors between two repeated assays ((**a**) and (**c**)) or duplicate wells ((**b**) and (**d**)) and the horizontal dashed line represents the blocking titer 50% (BT50).

To study the effect of amino acid 310 mutation on cross-blocking activity we used GII.4 1999 pooled immune sera to block wild-type GII.4 2012 and mutated GII.4 2012 D310N VLP binding to type A saliva (Figure 4a), PGM (Figure 4b), and synthetic H-type-1 (Figure 4c). In saliva A and H-type-1 blocking assays BT50 increased 2-fold (from 1:40 to 1:80, Figure 4a and from 1:80 to 1:160, Figure 4c), and in PGM blocking assay 4-fold (from 1:40 to 1:160, Figure 4b) in favor of the mutated VLPs. We also investigated the sera blocking activity of GII.4 2012-immunized mice against GII.4 2012 D310N mutant in synthetic H-type-1 blocking assay (Figure 4d) and no differences were observed in blocking of wild-type and mutated GII.4 2012 VLPs.

### 3.5. T-Cell Responses

**Figure 5:**
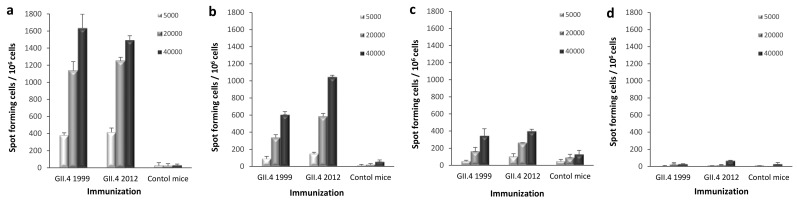
Norovirus (NoV) GII.4-specific T-cell responses. Group-wise pooled splenocytes of mice immunized with GII.4 1999 virus-like particles (VLPs), GII.4 2012 VLPs, or PBS only (control mice) were analyzed for interferon gamma (IFN-γ) production by an ELISPOT assay. Increasing number (legend) of bone marrow-derived dendritic cells (BMDCs) pulsed with GII.4 1999 (**a**), GII.4 2012 (**b**), or GII.4 2006 (**c**) VLPs were used to stimulate the splenocytes of GII.4 1999 or GII.4 2012 VLP-immunized mice. Un-pulsed BMDCs (**d**) served as a negative control. Mean IFN-γ spot-forming cells per 10^6^ live splenocytes of duplicate wells with standard deviations (error bars) are shown.

ELISPOT IFN-γ was used to investigate whether there are differences in the development of homotypic or heterotypic T-cell immunity after GII.4 1999 and GII.4 2012 VLP immunization. IFN-γ-producing cells were quantified from mice splenocytes in response to autologous BMDCs pulsed with different NoV GII.4 VLPs. GII.4 1999 or GII.4 2012 VLP-immunized mice splenocytes responded with similar intensity to GII.4 1999 (Figure 5a), GII.4 2012 (Figure 5b) and GII.4 2006 VLP (Figure 5c) pulsed BMDCs used as APCs in the ELISPOT. The highest IFN-γ production was induced with GII.4 1999 VLP pulsed APCs followed by GII.4 2012 and GII.4 2006 VLP pulsed cells. The magnitude of IFN-γ releasing cells increased with higher number of pulsed BMDCs. No significant IFN-γ production was detected when un-pulsed BMDCs were used as a negative control (Figure 5d). All samples were tested in duplicate cells. Cell viability of the responding cells was similar in all assays as controlled by Con A stimulation (data not shown). Background control (splenocytes in CM only) resulted in <40 spots per 10^6^ cells.

## 4. Discussion

**Figure 6:**
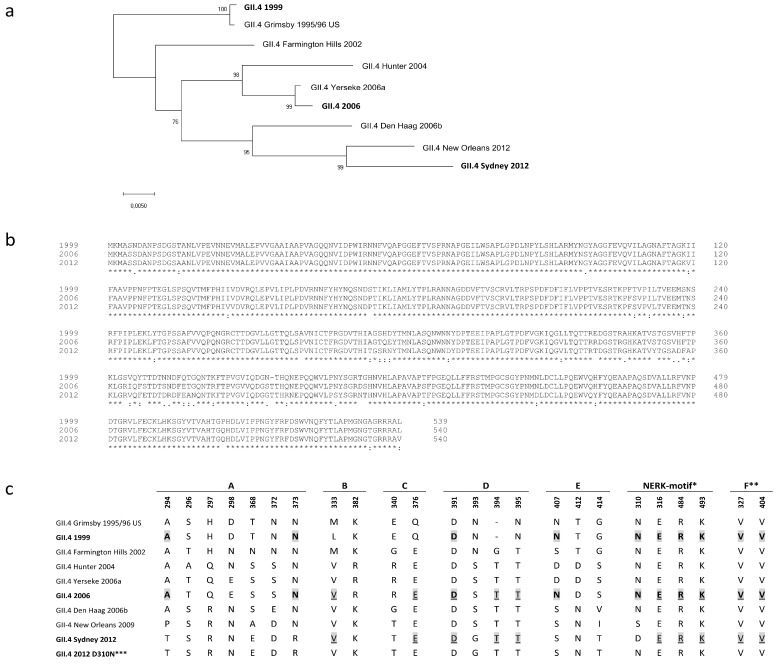
Phylogenetic tree and the amino acid (aa) sequences within blocking epitopes A–F and the NERK motif based on the VP1 amino acid sequences of norovirus (NoV) GII.4 strains. The tree illustrates the phylogenetic analysis of seven pandemic GII.4 NoV major capsid protein VP1 aa sequences with the GII.4 variant VP1 sequences used as virus-like particles (VLPs) in this study (boldface) (**a**). The tree was inferred by neighbor-joining method with a bootstrap of 1000 by using MegaX. The percentages of replicate trees in which the associated taxa clustered together in the bootstrap test (1000 replicates) are shown next to the branches. The evolutionary distances were computed using the p-distance method and the scale bar shows the genetic distance, expressed as amino acid substitutions per site. ClustalW VP1 amino acid sequence alignment of GII.4 1999, GII.4 2006, and GII.4 2012 variants used as VLPs in this study (**b**). Asterisk (*) indicates fully conserved amino acids, colon (:) indicates conservation between groups of strongly similar properties and period (.) indicates conservation between groups of weakly similar properties. Amino acid sequences of blockade epitopes A to F and the NERK motif [21,25,31] in GII.4 variants (**c**). Identical amino acids between GII.4 1999 and GII.4 2006 are shaded and bolded. Identical amino acids between GII.4 2012 and GII.4 2006 are shaded and underlined. *Motif regulating antibody access to the epitope F [31]. **Critical key residues of blockade epitope F [25]. ***Mutated VLP used in this study. Genbank accession numbers to VP1 amino acid sequences of published GII.4 strains are as follows: AF080551 (1995/96 US), AY502023 (Farmington Hills 2002), DQ078794 (Hunter 2004), EF126963 (Yerseke 2006a), BAG70446 (2006), EF126965 (Den Haag 2006b), GU445325 (New Orleans 2009), AFV08795.1 (Sydney 2012).

An important issue in NoV VLP vaccine development is the antigenic diversity of NoV genotypes and the evolution of the predominant GII.4 strain resulting in variants able to escape herd immunity [5]. A similar phenomenon drives the antigenic drift of influenza virus, and therefore influenza vaccine must be reformulated on a yearly basis to match the circulating strains [16,46]. The NoV VLP vaccine might also need to be reformulated every few years, or else cross-blocking epitopes could be induced to generate broadly blocking antibodies that protect against a variety of NoV variants [28,30,47]. In our earlier studies we have shown that ancestral GII.4 1999 VLPs tend to induce immune responses with better cross-reactivity and higher quality than other NoV genotype VLPs [48,49,50]. To further investigate GII.4 1999 VLP potential as a vaccine antigen, we compared the immune responses induced by GII.4 1999 VLPs and the most recent pandemic variant GII.4 2012 VLPs in mice. GII.4 2006 VLPs, representing pandemic variant 2006a, were selected as a heterologous GII.4 antigen as the genetic distances to GII.4 1999 and GII.4 2012 variants were approximately equal (Figure 6a), with VP1 sequence amino acid identities of 94.4% and 94.1%, respectively (Figure 6b).

Avidity of antibodies is considered to be an important surrogate of protective efficacy of several vaccines [51,52] and high avidity enhances the cross-reactivity of antibodies by tolerating minor variation in the target epitopes [53]. In this study, only GII.4 1999 VLPs were able to induce type-specific antibodies with high avidity, whereas GII.4 2012 VLPs induced only antibodies with very low avidity. B cells that take up an antigen can either mature into extrafollicular plasmablasts secreting low-affinity antibodies or enter into germinal center (GC) where affinity maturation takes place with the help of follicular DCs and T-cells [54]. GII.4 1999 and GII.4 2012 VLPs may be differently uptaken or presented in GC affecting affinity maturation, but further studies are needed to confirm this notion. However, based on the results obtained here, antibody avidity cannot be considered as a single correlate of a strong blocking activity, as GII.4 2012 VLPs induced poor avidity antibodies but still conferred homologous blocking similarly to GII.4 1999 serum. Instead, high antiserum avidity may enhance the cross-neutralization ability as broadly neutralizing antiviral antibodies are usually detected in recurrent/chronic infections or after repeated vaccinations [55].

We observed that GII.4 1999 immune serum had blocking activity against GII.4 2012 but not vice versa. The finding is in concordance with our earlier results where GII.4 1999 immune serum was able to block the VLP binding of another contemporary variant (GII.4 NO) to HBGAs whereas GII.4 NO immune serum completely lacked blocking activity against GII.4 1999 [48]. There might be several reasons as to why an ancestral GII.4 variant would induce blocking antibodies against a contemporary GII.4 variant but not vice versa. One potential explanation could be the surface structure of these VLPs. X-ray crystallography with murine NoVs has revealed that P-domains can be either in “open” or “closed” conformation [28] and some NoV VLPs are found in more epitope-accessible form than others [25], giving reason to speculate as to whether such structural differences affect the immune responses generated in vivo. Hypothetically, better epitope-accessibility promotes the development of broadly blocking antibodies as conserved blocking epitopes tend to locate in more occluded than exposed parts of the NoV capsid [25,47] and thus are not easily reached by B cell receptors. GII.4 2012 VLPs might be in a less epitope-accessible form than GII.4 1999 VLPs, explaining the different levels of humoral cross-reactivity induced. Possibly the evolution drives NoV to alter its structure to a more closed one to sterically protect the conserved epitopes, and therefore ancestral strains might be more suitable for the generation of cross-reactive immunity. However, additional studies are needed to evaluate the conformational differences and the epitope-accessibility between GII.4 variants.

GII.4 1999 immune serum cross-blocked GII.4 2006 VLP binding similarly to homologous VLP, whereas GII.4 2012 serum cross-blocking activity against GII.4 2006 VLPs was significantly lower. Other investigators have bioinformatically identified blocking epitopes A-F and shown that evolution in these epitopes, especially in evolving epitopes A and D, might have resulted GII.4 variants to escape from herd immunity [23,26,31]. Alignment of amino acid sequences of VLPs used in this study according to epitopes A–F and the NERK motif revealed that GII.4 1999 and GII.4 2012, together with GII.4 2006 variants, share the same number (10/22) of identical amino acids in these epitopes (Figure 6c). Epitope A holds 2/7 identical residues between GII.4 1999 and GII.4 2006, whereas none of the amino acids in the epitope A are shared between GII.4 2012 and GII.4 2006 variants. In contrast, GII.4 1999 and 2006 share only 1/4 amino acids in blocking epitope D, whereas 3/4 identical residues are found between GII.4 2012 and GII.4 2006 variants. Therefore, it is likely that the number of identical amino acids in the blocking epitopes A–F does not solely explain the better GII.4 2006 VLP cross-blocking potency of GII.4 1999 immune serum. However, some amino acids, such as 294 and 373 in the epitope A [23,26] and 310 in the NERK motif [31], shared between GII.4 1999 and GII.4 2006 VLPs might have greater impact on cross-blocking responses than other amino acids. In addition to blocking epitopes discussed here, other epitopes affecting the blocking responses have been published [28,29] and there could be other yet undiscovered regions impacting the cross-blocking responses.

Broadly conserved epitopes are often occluded in the capsid and can be shielded by the virion via selected motifs regulating the exposition of these epitopes [27]. Epitope F in NoV GII.4 is an occluded, broadly conserved GII.4 blocking epitope, and the NERK motif is a potential conformational determinant regulating antibody access to epitope F [28,31]. The NERK motif has remained unchanged among pandemic GII.4 variants from 1995 to 2006 and thereafter evolved from SERK (GII.4 NO 2009) to DERK (GII.4 Sydney 2012) by a single amino acid substitution at position 310 (Figure 6c). Both GII.4 1999 and GII.4 2006 share asparagine (N) at position 310 and we reasoned that residue 310 might explain the great blocking activity observed between GII.4 1999 sera and GII.4 2006 VLPs. As a result, amino acid 310 substitution from D to N in GII.4 2012 VLP capsid improved GII.4 1999 immune serum blocking potency 2–4-fold. This moderate improvement in the cross-blocking potency might indicate that asparagine at position 310 limits the antibody access to blocking epitope less than aspartic acid (D) at the same position. Others have exchanged residue 310 between GII.4 NO and GII.4 2012 VLPs and the effect on blocking activity of monoclonal antibody targeted to blocking epitope F was investigated [31]. As a result, mutated GII.4 NO (S310D) VLP blocking decreased (at max 4.1-fold) and in turn mutated GII.4 2012 (D310S) blocking increased (at max 3.2-fold) suggesting that serine (S) at position 310 indicates better access to the conserved blocking epitope F [31]. The difference with respect to our study was that we used polyclonal mouse serum instead of a monoclonal antibody generated against the single particular epitope. In contrast to cross-blocking activity, the D310N mutation did not have any effect on the homologous blocking activity of GII.4 2012 immune sera. This indicates that NERK motif-“guarded” F epitope is important specifically in cross-blocking responses, which supports earlier findings by others [30,31].

The role of T-cell immunity in protection against NoV infection and disease is not well known but it might have a role in the generation of heterologous immunity [56,57]. We and others have previously identified conserved NoV-specific CD4+ and CD8+ T-cell epitopes in mice [36,49,58]. Here, we were interested to see if the different potentials of GII.4 1999 and GII.4 2012 VLPs to induce cross-reactive antibodies were true also for T-cell responses. However, we did not detect a considerable difference in T-cell responses against any of the VLPs between GII.4 1999- and GII.4 2012-immunized mice. The results indicate that in the light of cross-reactivity, T-cell responses are quite distinct from B-cell responses and support earlier findings that T-cell responses are targeted to buried, broadly conserved epitopes in the NoV capsid [36,49,56,58]. However, variation in the magnitude of the IFN-γ SFC between VLPs used for pulsing was detected. The highest number of IFN-γ-producing cells were detected with BMDCs pulsed with GII.4 1999 VLPs (regardless of the immunization), which suggests that GII.4 1999 VLPs could be uptaken and/or processed most efficiently by APCs.

We have previously proposed a candidate NoV VLP vaccine including GII.4 1999 and GI.3 VLPs [12]. The results of this study showed that GII.4 1999 VLP immunization induced higher-affinity antibodies with improved cross-reactive and cross-blocking properties in comparison to GII.4 2012 VLPs. The ability to elicit broad cross-reactive immunity is a key element of a successful NoV vaccine. We suggest that VLP structure (e.g., epitope accessibility), avidity of the antibodies and T-cell immunity might all play important role in heterologous NoV immunity and therefore should be considered in vaccine VLP selection. Based on the results of this study, and our earlier findings [48,49,50], the ancestor GII.4 1999 VLPs have an intrinsic property of inducing antibodies with broad cross-reactivity and thus are good candidates for an NoV VLP vaccine.
